# Are female bipolar patients of reproductive age aware of the teratogenic risk of sodium valproate? A qualitative study

**DOI:** 10.4102/sajpsychiatry.v28i0.1719

**Published:** 2022-01-31

**Authors:** Amanda U. Sibanyoni, Marinda Joubert, Kalaivani Naidu

**Affiliations:** 1 Department of Psychiatry, Faculty of Medicine, University of Pretoria, Pretoria, South Africa

**Keywords:** sodium valproate (epilim), teratogenicity, bipolar disorder, childbearing age, 18–44 year olds

## Abstract

**Background:**

Sodium valproate is considered the most teratogenic of all anticonvulsant drugs. Internationally, new regulations require women to sign risk assessment forms if initiated on it.

**Aim:**

This study aimed to explore patients’ awareness of the teratogenic risk of sodium valproate.

**Setting:**

Weskoppies Psychiatric Hospital, Tshwane, Gauteng.

**Methods:**

We conducted a qualitative study comprising 23 semi-structured interviews with female bipolar patients of reproductive age at a tertiary psychiatric hospital in South Africa.

**Results:**

Patient psychoeducation and self-education is improving as many patients were aware of the risk of teratogenicity of sodium valproate either by being educated or by searching online after developing an interest. Our study identified the need for female patients to be educated about contraceptive use when starting on sodium valproate to avoid pregnancy.

**Conclusion:**

Our study shows that patients are becoming more aware of the teratogenic risk of sodium valproate. This suggests that consultations focusing on the issues of conception and the use of sodium valproate in women of childbearing potential has improved.

## Introduction

Bipolar disorder affects approximately 1% – 2% of the population.^1,2^ While men and women are similarly affected, several aspects of bipolar disorder require unique consideration in women. Of particular concern for women is how a condition may affect their reproductive function, and how they should be treated if they are planning pregnancy, are pregnant, postpartum or breastfeeding. The reproductive health of female psychiatric patients has been neglected in the field of psychiatry.^1,2,3^

The National Institute for Health and Care Excellence (NICE) recommends sodium valproate as a second line treatment for preventing relapse in bipolar disorder. There is also some evidence for efficacy in the treatment of mania and bipolar depression.^4^ The American Psychiatric Association also recommends using sodium valproate to manage acute mixed or manic episodes and as a maintenance treatment of bipolar I disorder.^5^

Despite the treatment benefits, healthcare providers are becoming more aware of the risks associated with prenatal exposure to sodium valproate. Internationally, recommendations are being updated with restrictions to sodium valproate use and for providing enhanced pre-conception counselling for women of childbearing age.^6,7^ In-utero exposure to sodium valproate during the first three months of pregnancy may result in foetal-valproate syndrome.^8^ This disorder has a spectrum of recognisable abnormalities including some common facial dysmorphic features and minor skeletal abnormalities. Other teratogenic risks include cardiovascular, craniofacial, urogenital, digital, and respiratory tract abnormalities and developmental delay. There is also an increased risk for miscarriage or stillbirth.^9^ In-utero exposure to sodium valproate has also been associated with a reduction in intelligence quotient (IQ) by an average of nine points and a fivefold higher risk of developing childhood autism.^4,10^ The risk of major congenital malformations is increased with the use of higher doses of sodium valproate. An observational study by Thomson et al. found a higher incidence in patients who were on a dose higher than 1500 mg/day as compared to lower doses.^11^

Given the risks for women of childbearing age, women with bipolar disorder should be counselled about the potential side effects of sodium valproate and the importance of contraception. Surprisingly, counselling is not commonly offered even though bipolar disorder is associated with impulsive sexual behaviour and medications may be unsafe during pregnancy.^12,13^ The use of sodium valproate in the general population is increasing, especially amongst women of childbearing potential.^7^ Despite sodium valproate being increasingly prescribed, the rate of clinical discussion around the teratogenic risk and pregnancy planning has declined.^6,7^ The co-prescription of folic acid or contraceptives with sodium valproate has also declined over the years.^14^

Guidelines produced by the Royal College of Obstetricians and Gynaecologists in 2016 recommended that any woman of childbearing potential should avoid sodium valproate reflecting a previous NICE guideline published in 2014.^7^ More recently, regulatory bodies have tightened their stance considerably. In February 2018, for example, the Pharmacovigilance Risk Assessment Committee of the European Medicines Agency recommended that sodium valproate should not be prescribed to women of childbearing potential who are not on a pregnancy prevention plan.^7,15^ This programme aims to ensure that all female patients on sodium valproate have been educated about the risks of taking the drug, have signed a Risk Acknowledgement Form, are on highly effective contraception and see their specialist at least once a year.^16,17^ This is especially important as valproate is now considered the most teratogenic of all anticonvulsant drugs.^6,18^ Ideally, sodium valproate should not be offered to women of childbearing potential for the management of bipolar disorder whether acute or long-term, and if already prescribed, it should gradually be discontinued because of its teratogenic risk.^19,20^

In light of the new regulations requiring a woman of childbearing potential to sign a risk acceptance form if she chooses to continue valproate, informed decision-making is imperative. The use of sodium valproate during pregnancy remains a matter for debate and there is scope for further research into the relative efficacy and safety of alternative treatments, as well as the dynamics of communication and shared decision-making in this situation.^7,21,22^ To our knowledge, another study was found dealing with this topic. This study was performed in Ireland by Mulryan et al who looked at the patients’ awareness of the teratogenic effects of valproate. The study also aimed to evaluate clinical note documentation of valproate prescribing and to establish the level of knowledge that these women had. The study found little documentation of patient awareness and education in their clinical files and only a small percentage of patients described this awareness.^23^ This highlights the failure in adequate education of this patient group in healthcare systems today.

## Methods

### Study design

This qualitative study was conducted at Weskoppies Psychiatric Hospital, a tertiary psychiatric hospital in the Tshwane District of the Gauteng province in South Africa.

### Study population and sampling

We interviewed 23 female mental healthcare users diagnosed with bipolar I disorder on sodium valproate in once off, semi-structured interviews. The study participants were purposively sampled.

### Inclusion and exclusion criteria

We included women between the ages of 18 and 44 years, and who had childbearing potential, as defined by the Centers for Disease Control and Prevention (CDC). The patients were either admitted to hospital or managed at the outpatient department between May and September 2019.

The patients were stabilised and in partial or full remission. All the outpatients (16) were on a continuous, maintained dose whereas two of the seven inpatients were still undergoing titration of their medication. The patients were on various doses of sodium valproate ranging from 800 mg to 2.4 g daily. Patients were not only on sodium valproate. About 20 of the 23 participants were also on an antipsychotic. Five were also on antiretroviral treatment.

We excluded potential participants if they were unable to give informed consent, if they suffered from substance intoxication in addition to bipolar I disorder, who were unstable in manic or depressive phase of illness, who were psychotic as well as those who had taken a benzodiazepine 24 h before the interview. We also excluded patients who did not comply with the study requirements and those taking valproate primarily for epilepsy and postmenopausal patients.

### Data collection

The interviews were conducted in English, Setswana and isiZulu. The interviews were semi-structured with open-ended questions. Basic demographic data were captured in a data collection sheet (see Table 1). The initial questions were designed to stimulate discussion, whilst adding structure. The initial questions were based on the researchers’ perceptions of what it would take to get the interview started. The participants were encouraged to elaborate freely on every question allowing them to share their knowledge and experiences. Most interviews were concluded within 20 min. Field notes were taken during the interviews. Each interview was audio recorded and then transcribed for further analysis.

**TABLE 1:** Demographic data of 23 female bipolar patients who were interviewed regarding their perceived awareness of teratogenic risks associated with using sodium valproate.

Demographic	Value
**Age**	
18–25	4
26–35	13
36–45	6
**Marital status (*n* = 23)**	
Married	4
In a steady relationship	14
Single	5
**Number of children**	
No children	10
One child	8
Two children	3
Three children	2
Expressed intent to have more children	16
Residential area	The area highly represented in the study was central western townships of Pretoria, that is, Atterigdgeville and Saulsville

Data from 23 interviews were included. All participants gave written informed consent. No names or identifying data of mental healthcare users were recorded or used when reporting the results.

The interview transcriptions and field notes were analysed using thematic content analysis in which a coding scheme was used to identify the most common or recurrent themes that emerged from the data. After 14 interviews were conducted, recurrent themes started to emerge, thus indicating data saturation.

### Ethical considerations

The study was approved by the University of Pretoria, Faculty of Health Sciences Ethics Committee, reference no: 59/2019.

## Results

From the patient interviews, we identified three themes, each with subthemes. The three themes included knowledge about teratogenicity, counselling about teratogenicity and concerns about being on sodium valproate.

### Theme 1: Knowledge about teratogenicity

Subthemes:

1.1 Knew nothing at all about sodium valproate and teratogenicity
1.2 Had a broad idea/s about teratogenicity
1.3 Had knowledge regarding teratogenicity
1.4 Knowledge regarding steps to follow if planning a pregnancy or if they fall pregnant whilst on valproate

#### Subtheme 1.1

Four of the 23 participants had no knowledge regarding the teratogenic effects of sodium valproate. The participants reported being on sodium valproate for long periods at the hospital outpatient department, district hospitals or local clinics but knew nothing regarding teratogenicity as a possible side effect.

‘I have never been educated about epilim. It is the first time I hear about epilim and pregnancy today. I don’t know any risks.’ (Participant A4, 33, female)

‘I can’t remember but they didn’t say it is dangerous. They just said to eat before taking it; they didn’t talk about side effects.’ (Participant A13, 38, female)

#### Subtheme 1.2

Six of the 23 participants had an idea or were suspicious about the risks of sodium valproate to an unborn child. They knew it was not safe to take sodium valproate during pregnancy. It was clear that this group acquired this knowledge informally from friends, associates, and so on and they had no formal counselling:

‘I also believe the baby feels pain when the mother drinks epilim. Especially if the dose is too high. I take 1000 mg three times per day so that cannot be good for the baby as well. It will make the baby drowsy in the womb. The baby can also have abnormalities such as eyesight problems, problems in the joints, limb deformities.’ (Participant A17, 32, female)

‘I have heard people say if I have bipolar then I drink epilim, the baby might also have bipolar. I don’t know about side effects in an unborn child.’ (Participant A22, 33, female)

#### Subtheme 1.3

Most of the participants, 13 of the 23, knew about the teratogenic effects of sodium valproate. They were aware of this from being psycho-educated or counselled by healthcare workers or by discovering the information on their own:

‘Yes I am aware of the risks; if someone is pregnant and they take epilim the baby may become abnormal. Epilim can cause the baby to drool, to be cripple, to have mental problems, to be a slow learner.’ (Participant A8, 29, female)

‘The child will be deformed; the spinal cord will be abnormal. The baby can also have imbalances in the brain and have madness and the baby can have speech problems or have incomplete limbs. The baby can be a slow learner when they grow up.’ (Participant A18, 27, female)

#### Subtheme 1.4

Inclusive of all the participants, regardless of their knowledge of teratogenicity, 20 of the total number of 23 knew there were steps to follow should they be planning a pregnancy or if they fall pregnant whilst on epilim.

‘You must go to the Dr and tell her you want a baby. They will put you on other medicine.’ (Participant A5, 23, female)

‘Yes, I am aware that I need to inform doctors because they would need to adjust medication in order for me to fall pregnant.’(Participant A12, 31, female)

### Theme 2: Counselling regarding teratogenicity

Subthemes:

2.1 Counselling received at Weskoppies Hospital
2.2 Counselling received at the local clinic or other psychiatric hospitals
2.3 No counselling received at district level hospitals
2.4 Searched online for teratogenicity in sodium valproate after deriving an interest
2.5 Counselling regarding contraceptives
2.6 Information shared during counselling

#### Subtheme 2.1

Nine of the 13 participants who knew about teratogenicity were counselled at Weskoppies Hospital as inpatients by their treating doctors, nursing staff or other members of the multidisciplinary team:

‘In 2014 after I had my baby. Here in Weskoppies they told me about epilim …Yes, social workers, doctors and nurses would teach us.’ (Participant A11, 37, female)

‘Yes I got counselling when I started epilim and yes I then started the injection. The last two years … from Weskoppies.’ (Participant A12, 31, female)

#### Subtheme 2.2

Four of the 13 participants who knew about teratogenicity had been counselled at other psychiatric hospitals prior to being transferred to Weskoppies Hospital. Other participants, who would follow up at their local clinic, received counselling there:

‘I am aware of the risks but I don’t know what it is. When I fell pregnant in 2012 I was told that because I am drinking epilim there are risks … the baby will be born with poor eyesight or complications in the brain or something like that … I was told by the nurses at the clinic.’ (Participant A2, 29, female)

‘I was told your baby may grow without a bone structure I am actually from Cape Town and I attended Lentegeur Psychiatric Hospital in Mitchell’s Plain.’ (Participant A9, 44, female)

#### Subtheme 2.3

Two of the participants who had been referred to Weskoppies Hospital from district level hospitals reported not receiving counselling at their district hospital. One participant reported that no counselling is conducted at district hospitals:

‘My friend told me that if you want to have a baby you should consult your physician, I was attending Mamelodi Hospital. I was on lithium before epilim. They told me epilim is for my mood but I was not told about side effects in a baby.’ (Participant A3, 38, female)

‘I was attending the hospital in Tshwane. No, they do not teach there. They only give you your medication. I relapsed and they sent me here. Now I collect my medication here.’ (Participant A6, 29, female)

#### Subtheme 2.4

Two participants were interested in learning more about sodium valproate, including its side effect profile because they had experienced other side effects from sodium valproate. Therefore, they did their own research to learn more about the drug:

‘That there are risks involved when taking epilim. I googled the information from the internet … I was gaining a lot of weight and then I was checking the side effects of the medication, then I found that when you are pregnant it is not safe to use epilim … They said the child might have defects in the brain; they might also have the same disease.’ (Participant A14, 44, female)

‘Yes. Epilim is very dangerous for the foetus in the sense that you can give birth to a deformed child. They can have defects. This is my own information actually. I think I am wrong. It cannot do anything to a child. The stuff I am saying is from Google and I can’t trust Google.’ (Participant A24, 33, female)

#### Subtheme 2.5

Fourteen of the 23 participants reported being on contraceptives. Nine of the 14 participants were on contraceptives for reasons not related to the side effects of sodium valproate. They stated that they do not want any more children because they were unemployed and financially constrained. The participants reported the need to take care of their mental health and not able to manage children. Some participants were advised to be on contraceptives for family planning purposes. Only five participants were on contraceptives to prevent teratogenicity if they were to fall pregnant on valproate:

‘It is because I am not financially stable; I realised a baby costs a lot of money and I don’t want another one.’ (Participant A4, 33, female)

‘The baby can have deformities … Yes I got the counselling when I started epilim, I was then also started on the injection.’ (Participant A12, 31, female)

‘Yes I do get an injection in the ward; it is so that I do not have another child, because I am sick.’ (Participant A21, 36, female)

#### Subtheme 2.6

Most participants shared similar knowledge after counselling or psychoeducation suggesting that clinicians shared similar information, which can be improved. Information sharing remains quite vague with some patients lacking understanding and the information being lost in translation.

‘I was told epilim is dangerous, it will damage the child. The baby will be born damaged or with deformities.’ (Participant A20, 36, female)

‘The child will be like those children that can’t talk, they can be crippled, the child will not move in the womb.’ (Participant A23, 29, female)

### Theme 3: Concern about being on valproate

Subthemes:

3.1 Raised concern about being on sodium valproate because of side effects experienced or concerns regarding teratogenicity
3.2 Believed sodium valproate may have negatively affected previous pregnancies or plans to conceive and some were not happy about sodium valproate as the drug of choice

#### Subtheme 3.1

Three participants felt that sodium valproate is a bad and dangerous drug that they do not like to take and were not happy with it being the drug of choice. They believed that sodium valproate causes severe side effects for themselves and to the potential unborn children:

‘Epilim is very bad for one because the thing is it gives you things like milk which is not milk. Sometimes it lets you stay awake at night. So it is not good, epilim is not good. It can also cause hair loss. There is extensive hair loss in epilim and the other thing that it give, feels like an electrical shock for your brain at times. I believe epilim is not good for the baby because the thing is the medication goes from one to another.’ (Participant A10, 43, female)

‘[*B*]ut it can affect the liver- that is a side effect. It is like you are faced between the red sea and the devil you have to choose one. I have to take epilim until I die but it can mess up everything. It is very dangerous … It can make a baby psychotic.’ (Participant A16, 26, female)

#### Subtheme 3.2

Two participants reported having complications in previous pregnancies, three reported being unable to have more children even though they would have liked to. These issues were believed to have been caused by being on sodium valproate at the time. Some participants also told that they were unhappy about being on sodium valproate as the drug of choice:

‘Other people are telling me if you are using epilim you will not catch up. I do not know what to believe. I am not using contraceptives, I am ready to catch up and I am not. They told me to stop using epilim so that I can fall pregnant.’ (Participant A7, 44, female)

‘Yes I would have probably have had another baby already because I am very maternal but I am on epilim and asked them is there anyway of me ever weaning myself off the epilim?’ (Participant A9, 44, female)

‘It is very dangerous; I lost two kids to epilim. Last year I was pregnant, they gave me epilim, and olanzapine and I lost the babies. I was in hospital after taking epilim I bled for seven days. So what else could it have been but epilim? Epilim is a drug I detest. The reason I take it is to make the doctors feel good. I prefer olanzapine. It is a bit mild. The side effects are better. Epilim can destroy your liver … Epilim can affect the mental function of the child; the child can also be psychotic.’ (Participant A16, 26, female)

## Discussion

In this study, we qualitatively explored the awareness of female bipolar patients of reproductive age about the teratogenic effect of sodium valproate. Given the risks associated with using sodium valproate during pregnancy, it is important to understand how aware women of childbearing potential are regarding these risks and how much they know about what precautions they should be taking.

Internationally, the recommendations and restrictions for using sodium valproate are being updated and pre-conception counselling for women who plan to have children is encouraged. To our knowledge, this is the first such study in our setting. Compared to the study by Mulryan et al which showed that about 33% of the study participants described as having an awareness of these risks from consultation with their treating mental health team^23^, 56% of the participants in our study knew about the teratogenicity associated with sodium valproate either via psychoeducation from their treating psychiatrists, doctors, nurses or by obtaining information from the internet. Of the total number of participants who had the knowledge of teratogenicity of sodium valproate, 84% were informed by their treating psychiatrists or doctors. Our results indicate that patients are being psycho-educated at regional hospitals, but counselling at district level hospitals should be enhanced.

Our results indicated that the mode of information delivery should also be enhanced. A study by James et al. showed that few prescribers demonstrated good awareness of the estimated teratogenic potential of the drug and practice was characterised by a reluctance to discuss contraception with patient.^24^ Our participants indicated that the clinicians seemed to share similar, vague and general information. The participants reported not understanding the information, which was sometimes lost in translation. Psychiatrists, doctors, and nurses should be knowledgeable about the risks of valproate use, and be able to share this knowledge comfortably with patients.

In our study, lack of knowledge also caused other concerns regarding sodium valproate use amongst participants. Because the participants did not know about all the potential side effects and benefits of sodium valproate use, they felt that the drug was ‘bad and dangerous’. A few participants expressed that being on sodium valproate had prevented them from having more children and bigger families as they had wished to. Some expressed being unhappy with sodium valproate as the drug of choice because of its side effect profile. After the interviews, the participants were psycho-educated and referred to their treating healthcare workers for further management. This helped to alleviate anxiety associated with the teratogenic risk of sodium valproate.

In our study, most participants, 14 of the 23, were on contraceptives. They used contraceptives for different reasons. These reasons included not wanting any more children, financial constraints and not being physically able to care for any more children. Only five participants were advised to avoid falling pregnant whilst on sodium valproate. They had also been educated regarding teratogenicity and contraceptive use in sodium valproate. Our findings indicate that doctors and nursing staff are not adequately educating patients regarding the use of contraceptives and sodium valproate, nor are they prescribing contraceptive medication when initiating sodium valproate, which is the standard practice. Although most of our patients knew about teratogenicity and sodium valproate, they did not seem to know about contraceptive use when on sodium valproate. Although most participants were on contraceptives, it was mainly for other reasons. Comparatively a study by Langan et al. showed low rates of teratogenic risk counselling and low rates of contraception advice in the cohort.^25^

Our study shows that psychoeducation in the form of patients being informed about the possibility of side effects of the drug whilst balancing or weighing against the magnitude of the disease is vitally important in this group of patients. It is also important to identify these patients early, to avoid prescribing sodium valproate if possible, and to switch to safer drugs early in pregnancy. Sodium valproate use in pregnancy is not recommended and appropriate prescribing should be practised in this patient group.^22,26,27^

### Limitations

This study has a number of limitations, including small sample size. This is to be expected because of the specificity of the sample population. The findings were attained from one hospital (region) and may not be representative of other regions. Additional information from various settings would also be necessary to evaluate any trends. This is a qualitative study and therefore the findings have limited objectivity and statistical validity. The semi-structured interviews conducted were retrospective in nature, and thus potentially liable to recall bias. The generalisability of this study results are limited, because of its thematic interpretation. The teratogenic effect of sodium valproate is dose-dependent and the dose of sodium valproate was not scrutinised in this study.

## Conclusion

Our study shows that patients are becoming more aware of the teratogenic risk of sodium valproate. This suggests that consultations focusing on the issues of conception and the use of sodium valproate in women of childbearing potential has improved. We, however, found that there is a lack of quality information and detailed knowledge on the subject of the teratogenic risk of sodium valproate.
